# King Edward's Hospital Fund for London

**Published:** 1921-03-19

**Authors:** 


### King Edward's Hospital Fund for London.

We are asked to state that hospitals in the county of London or within nine miles of Charing Cross desiring to participate in the grants made by this Fund for the year 1921 must make application before March 31 to the Hon. Secretaries, 7 Walbrook, E.C. 4. Applications will also be considered from Convalescent Homes which are situated within the above boundaries, or which, being situated outside, take a large proportion of patients from London; and from sanatoria for consumption which take patients from London, or which are prepared to place beds at the disposal of the Fund for the use of patients from London hospitals.

